# Identification of a Potential Regulatory Variant for Colorectal Cancer Risk Mapping to 3p21.31 in Chinese Population

**DOI:** 10.1038/srep25194

**Published:** 2016-04-28

**Authors:** Juntao Ke, Jiao Lou, Rong Zhong, Xueqin Chen, Jiaoyuan Li, Cheng Liu, Yajie Gong, Yang Yang, Ying Zhu, Yi Zhang, Jiang Chang, Jing Gong

**Affiliations:** 1 State Key Laboratory of Environment Health (Incubation), MOE (Ministry of Education) Key Laboratory of Environment & Health, Ministry of Environmental Protection Key Laboratory of Environment and Health (Wuhan), School of Public Health, Tongji Medical College, Huazhong University of Science and Technology, Wuhan, China

## Abstract

Genome-wide association studies (GWAS) have established chromosome 3p21.31 as a susceptibility locus for colorectal cancer (CRC) that lacks replication and exploration in the Chinese population. We searched potentially functional single nucleotide polymorphisms (SNPs) in the linkage disequilibrium (LD) block of 3p21.31 with chromatin immunoprecipitation-sequencing (ChIP-seq) data of histone modification, and tested their association with CRC via a case-control study involving 767 cases and 1397 controls in stage 1 and 528 cases and 678 controls in stage 2. In addition to the tag SNP rs8180040 (odds ratio (OR) = 0.875, 95% confidence interval (95% CI) = 0.793−0.966, *P* = 0.008, *P-FDR* (false discovery rate) = 0.040), rs1076394 presented consistently significant associations with CRC risk at both stages with OR = 0.850 (95% CI = 0.771−0.938, *P* = 0.001, *P-FDR* = 0.005) under the additive model in combined analyses. Supported by the analyses of data from The Cancer Genome Atlas (TCGA) and Gene Expression Omnibus (GEO), it was suggested that rs1076394 served as an expression Quantitative Trait Loci (eQTL) for gene *CCDC12* and *NME6*, while *NME6*’s expression was obviously higher in CRC tissues. Using biofeature information such as ChIP-seq and RNA sequencing (RNA-seq) data might help researchers to interpret GWAS results and locate functional variants for diseases in the post-GWAS era.

Colorectal cancer (CRC) was the third most commonly diagnosed cancer in males and the second in females worldwide in 2012^1^, while it was the fifth in males and the third in females, with an estimated 310,244 new cases and 149,722 deaths in China in 2011^2^. Several environmental factors, including diet, physical inactivity, obesity, cigarette smoking and alcohol consumption, were indicated as being involved in the occurrence and development of CRC^3^,^4^,^5^. On the other hand, it has been well established that genetic factors play an important role in CRC etiology^6^,^7^,^8^. Revolutionary genome-wide association studies (GWAS) and subsequent fine mapping researches have positioned over 30 susceptibility loci of CRC in Europeans^9^,^10^,^11^,^12^,^13^,^14^,^15^,^16^,^17^,^18^,^19^ and Asians^20^,^21^,^22^,^23^, however most variants have been found to be only tag single nucleotide polymorphism (SNPs) residing in intergenic and intronic regions without a clear function.

A major challenge in the post-GWAS era is to identify the specific genetic variants that accounts for phenotype based on their functional biology^24^. Recent reports showed that regulatory genome elements can greatly help to identify these causal SNPs, which could exert an effect on gene expression by modulating the activity of promoters, enhancers, insulators and silencers^25^,^26^,^27^. Today, regulatory genomic regions are usually characterized by various histone modifications in the flanking nucleosomes^28^,^29^. For example, enhancers are typically marked by H3K4me1 (histone H3 monomethylated at lysine 4) and promoters by H3K4me3 (histone H3 trimethylated at lysine 4), and both are regarded as active when additionally marked by H3K27ac (histone H3 acetylated at lysine 27)^30^,^31^,^32^,^33^. And chromatin immunoprecipitation-sequencing (ChIP-seq) of histone modifications has been widely used to map genome-wide enhancers and promoters^34^,^35^,^36^,^37^.

Fernandez-Rozadilla *et al*. mapped 3p21.31 as a CRC-relevant genomic locus in a Spanish population with 2362 cases and 2517 controls^38^, with a pooled *P* = 2.163E-06 (odds ratio (OR) = 0.784, 95% confidence interval (95% CI) = 0.709–0.867) for the tag SNP rs8180040. Although it didn’t reach the common GWAS significance threshold of 10^−8^ and wasn’t included in the larger-scale genetic study by Zhang *et al*. in East Asians^23^, we considered this locus containing abundant genes as an attractive region to be researched in the Chinese population. Moreover, the strongest risk polymorphism rs8180040 was not in any known transcribed or regulatory sequences and not likely the causal SNP, which meant the real functional SNPs remained mined in 3p21.31.

Using epigenomic data obtained from relevant cell types represents a powerful approach to identifying functional SNPs in post-GWAS genetic researches^39^,^40^,^41^,^42^. In this study, we analyzed ChIP-seq data of histone modifications from CRC cell lines, searched common variants within the regulatory elements of the risk-associated locus 3p21.31, investigated their associations with CRC risk via a two-stage case-control study in the Chinese population, and tried to explain the underlying function by analyzing the data from The Cancer Genome Atlas (TCGA) and Gene Expression Omnibus (GEO). To the best of our knowledge, this is the first replication and exploration study on 3p21.31 in East Asians.

## Results

### Selection of Candidate SNPs

The LD block of GWAS susceptibility loci 3p21.31 was chromosome 3: 47035735-47452118. After a bioinformatics analysis (details in Methods), four common polymorphisms, rs2276854, rs807936, rs1076394 and rs807937, situated within the peaks of histone modification ChIP-Seq data generated from HCT116 or Caco2, were found in the above loci. These four SNPs were in high LD with each other (*r*^*2*^ > 0.9) according to the 1000 Genomes Project Phase 3 data of the CHB population. Among them, we saw rs1076394 as the most credibly functional variant due to its location in the overlapping region of H3k4me1 and H3k27ac ChIP-seq peaks (Table 1). The coexistence of these two histone modifications is broadly considered as a mark of active enhancer, while the appearance of either one of them is not. For the replication and exploration of 3p21.31, we genotyped the tag SNP rs8180040 and the potential regulatory SNP rs1076394 in Stage 1. We further validated the positive SNPs in another independent sample of Stage 2.

### Population Characteristics

Descriptive characteristics of the subjects in this study are detailed in Table 2. In both stages, no significant differences were found between patients and controls in the distribution of sex and age. As expected, significantly more smokers were presented in the cases than in the controls, given that cigarette smoking was a well-established risk factor for CRC^3^. And we did not see the same distribution in drinking status.

### Association Analysis

Both investigated polymorphisms, the presumably regulatory SNP rs1076394 and tag SNP rs8180040, were significantly associated with CRC risk in both stages and the combined analysis (Table 3).

In Stage 1, under a multivariable logistic regression model adjusted for gender, age, smoking and drinking status, individuals with AA genotype of rs1076394 had a significantly reduced risk of CRC (OR = 0.723, 95% CI = 0.559–0.936, *P* = 0.014, *P-FDR* (false discovery rate) = 0.035) compared to those with GG homozygotes. A dominant model was used to improve statistical power by combining the GA with AA into an A-carrier group (GA plus AA), and it showed that the allele A carriers had an obviously protective effect on CRC susceptibility (OR = 0.802, 95% CI = 0.669–0.963, *P* = 0.018, *P-FDR* = 0.03). Likewise, a positive outcome was found in the additive models, with a per-A-allele OR of 0.847 (95% CI = 0.748–0.960, *P* = 0.009, *P-FDR* = 0.045). As for rs8180040, we successfully replicated this GWAS tagSNP under the dominant model (OR = 0.811, 95% CI = 0.677–0.970, *P* = 0.022, *P-FDR* = 0.825) and additive model (OR = 0.861, 95% CI = 0.758–0.979, *P* = 0.022, *P-FDR* = 0.825) with nominal significance, but failed after FDR corrections.

Two promising variants were both further genotyped in the validation Stage 2. In agreement with the Stage 1, nominal significant associations were still exhibited between CRC risk and rs1076394 (dominant model: OR = 0.745, 95% CI = 0.584–0.951, *P* = 0.018, *P-FDR* = 0.09; additive model: OR = 0.833, 95% CI = 0.708–0.980, *P* = 0.027, *P-FDR* = 0.068), or rs8180040 (dominant model: OR = 0.747, 95% CI = 0.586–0.952, *P* = 0.018, *P-FDR* = 0.09; additive model: OR = 0.837, 95% CI = 0.713–0.983, *P* = 0.030, *P-FDR* = 0.075). When we combined two stages, positive results were still observed after FDR corrections (Table 3). And two polymorphisms were in high LD (r^2^ = 0.895) with each other in our total samples, similar to the data of 1000 Genomes Phase 3 of CHB (r^2^ = 1.000).

The results of interaction analysis between the promising SNP rs1076394 and smoking were detailed in Table S2, where no significant interactions were observed under either the multiplicative or the additive model in the two stages and the combined study.

### TCGA and GEO Data Analyses

**Figure 1:**
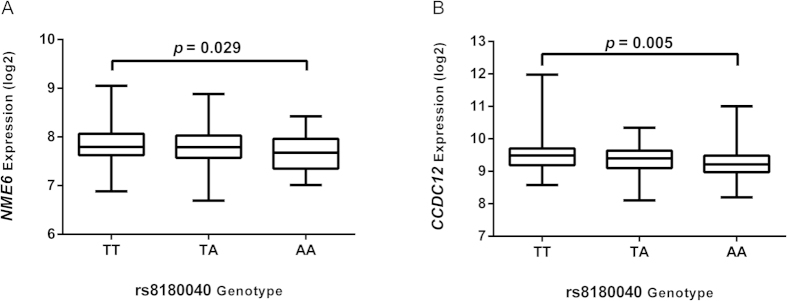
Association between rs8180040 and *NME6/CCDC12* expression. (**A**) Association between rs8180040 and *NME6* expression. (**B**) Association between rs8180040 and *CCDC12* expression.

We downloaded the data of gene expression, germline genotypes, CpG methylation and somatic copy number for COAD (colon adenocarcinoma) and READ (rectum adenocarcinoma) from the TCGA portal (http://cancergenome.nih.gov/) up to October 2014. Then, we performed a modified eQTL (expression Quantitative Trait Loci) analysis of the correlation between rs8180040 (rs1076394 wasn’t included in the Affymetrix GenomeWide SNP 6.0 Array of genotype profiles) and expression of genes within 1 Mb flanking regions, with the effects of somatic copy number and methylation being adjusted^43^. As shown in Table 4 and Fig. 1, rs8180040 was identified as an eQTL for genes *NME6* (nucleoside diphosphate kinase 6, R^2^ = 0.019, *P* = 0.029, *P-FDR* = 0.404) and *CCDC12* (coiled-coil domain-containing protein 12, R^2^ = 0.031, *P* = 0.005, *P-FDR* = 0.139), respectively. As the presumed regulatory variant rs1076394 was in complete LD with rs8180040 in the 1000 Genomes database, rs1076394 was also indicated as being closely related to the expression of *CCDC12* and *NME6*. In addition, we compared the two genes’ expression between cancer and normal tissue, and found a significant difference for *NME6* (*P* = 0.029; CRC tissues: 229.5 ± 3.2 RPKM (reads per kilobases per million reads), peritumoral tissue: 210.5 ± 5.4 RPKM), but not for *CCDC12* (*P* = 0.258; CRC tissues: 707.7 ± 15.3 RPKM, peritumoral tissue: 661.6 ± 16.49 RPKM).

In GEO database, the datasets of expression profiles in Asian samples (Dataset Records: GDS2609 and GDS4718) were provided, while TCGA data was mostly from Caucasian samples. We compared the *NME6* expression between cancerous and normal colon tissues, and found the same higher expression in cancer tissues from the Chinese population (*P* = 0.017; CRC tissues: 585.8 ± 29.3 RPKM, normal colon tissue: 482.2 ± 25.6 RPKM), and the Japanese population (*P* = 0.013; CRC tissues: 973.6 ± 79.9 RPKM, normal colon tissue: 771.4 ± 31.0 RPKM). As for CCDC12, we could not observe significant differences between cancer and normal tissues in two datasets (Chinese samples: *P* = 0.583, CRC tissues: 2219.9 ± 147.9 RPKM, normal colon tissue: 2118.0 ± 91.1 RPKM; Japanese samples: *P* = 0.062, CRC tissues: 22137.9 ± 971.7 RPKM, normal colon tissue: 24648.9 ± 870.9 RPKM).

## Discussion

The identification of tag SNPs through GWAS is the important first step in understanding the relationship between genomic variation and CRC risk. But now, the foremost goal in the post-GWAS era is to shed light on the causal SNPs and their functional consequences, progressing from indirectly statistical to directly biological associations between genetic variation and disease. Accumulating evidence showed that the most likely mechanistic basis that links those noncoding genetic variants to phenotype and disease is being regulatory^34^.

In our study, by overlapping the LD boundaries of the locus 3p21.31 and regulatory regions predicted by CRC-specific histone modifications, we screened out the most promisingly functional rs1076394 among the four original polymorphisms in high LD. By conducting association studies in two independent Chinese populations containing 1327 cases and 2075 controls in total, we replicated the significances of tag SNP rs8180040, and found a significant protective effect for the potentially regulatory variant rs1076394 that might serve as an eQTL for the genes *CCDC12* and *NME6*, while *NME6* presented significantly higher expression in cancer tissues.

The findings led us to assume that rs1076394 might influence CRC risk by altering the activity of an enhancer that controlled *NME6* expression. The potentially functional variant rs1076394 lay within a region of the genome exhibiting chromatin modifications H3k4me1 and H3k27ac in a CRC cell line HCT116, consistent with characteristics of an active enhancer across diverse tissues^44^,^45^. It was situated in the first intron of gene *KIF9* (kinesin family member 9), which was involved in mitotic progression by maintaining correct spindle length^46^, and the degradation of the matrix by regulating macrophage podosomes^47^. However, according to our calculation of TCGA data, rs1076394 was related to the expression of *CCDC12* and *NME6*, but not related to *KIF9*. We saw the SNP as a potential eQTL for *CCDC12* and *NME6*, while we could not rule out the possibility that it was actually in LD with these two genes. The SNP rs1076394 was approximately 300kb upstream of *CCDC12*, which participated in promoting early erythroid differentiation^48^, and over 1000 kb upstream of *NME6*, which might play a role in the regulation of cell growth and cell cycle progression^49^,^50^,^51^. Either *CCDC12* or *NME6* was located in 3p21.3. But, *NME6* was more suggested to be the actual contributing gene in this locus due to its significantly higher expression in cancer samples of both Caucasian and Asian populations. At the beginning, *NME6* was discovered as a gene encoding a nucleoside diphosphate kinase that suppressed p53-induced apoptosis^50^. In a shRNA functional screen, *Nme6* was reported to be crucial for the renewal of embryonic stem cells (ESCs). And ESCs are characterized by immortalization ability, pluripotency, and oncogenicity^52^. More recently, enriched somatic mutations in *NME6* was suggested to be associated with the deregulation of pyrimidine metabolism and the promotion of malignant progression in human melanoma^53^. Accordingly, *NME6* may act as an oncogene that still needs more investigations.

The application of biofeature information such as ChIP-seq and RNA-seq data has represented an effective approach to identifying functionally regulatory SNPs, and different databases including UCSC, Encode, GEO and TCGA have provided easy access to massive amounts of relevant data. Incorporating epigenetic and expression analyses into traditional molecular epidemiology could assist in the interpretation of GWAS results and the discovery of functional variants for diseases in post-GWAS studies. Using a similar strategy to other CRC-associated loci should deepen our understanding of CRC risk.

Nevertheless, several limitations should be noted here. First of all, due to the lack of relevant functional experiments, biological reality beneath the statistically significant association is uncertain. In the analysis of TCGA data, we have not restricted it to Chinese samples, when its composition is mostly Caucasian samples. It may not reflect the exact outcome of the Chinese population that we researched on. Second, the strategy of retrieving candidate polymorphisms depended on the prediction from ChIP-seq data of relevant cell lines, which was not rigorous enough to define exact regulatory elements and all the functional variants inside. Focusing on common SNPs, we could not rule out the possibility that sets of rare variants or haplotypes in LD with the tag SNP are actually causal in this locus. Third, insufficient epidemiological and clinical information prevented us from further investigating the interactions between gene and environment.

In summary, we discovered a probably regulatory SNP that was associated with CRC risk in the Chinese population. Researches on 3p21.31 and other CRC susceptibility loci with greater sample sizes and follow-up functional analyses are warranted to elaborate the biological mechanism of genetic etiology.

## Methods

### Study Participants

A two-stage case-control study was applied to evaluate the association between candidate variations and the risk of CRC. The discovery stage (Stage 1) consisted of 767 cases and 1397 controls, which were recruited from Tongji Hospital of Huazhong University of Science and Technology (HUST) between 2008 and 2012. The validation stage (Stage 2) involved 528 cases and 678 controls enrolled from 2013 to 2015 at the same hospital. All subjects were unrelated ethnic Han Chinese in both stages. Patients with histopathologically confirmed CRC and without previous chemotherapy or radiotherapy, were included without restriction to gender and age,. In the same time period, cancer-free controls were recruited form participants in physical examination programs of the same hospital, and were adequately matched to cases by gender and age (±5 years). Definitions of smoking and drinking status were the same as in a previous study by our group^54^,^55^,^56^. At recruitment of each subject, a written informed consent was obtained, and 2 millimeters peripheral venous blood was collected. This study was approved by the ethnics committee of Tongji Medical College of Huazhong University of Science and Technology, and the methods were carried out in accordance with the approved guidelines.

### SNP Selection and Genotyping

Candidate SNPs were common genetic variants (minor allele frequency, MAF > 0.05) that located in the putative regulatory elements of the 3p21.31 locus. First, we applied the software HaploView to calculate the linkage disequilibrium (LD) block of 3p21.31 with the criterion of *r*^*2*^ > 0.8, by inputting the Chinese Han Beijing (CHB) genotype information of 500 kb flanking the tagSNP rs8180040. This LD block was defined as the CRC susceptibility locus. Second, we downloaded ChIP-seq data regarding histone modification from two CRC cell lines, HCT116 and Caco2, form the UCSC database integrated with Encode data (S1 Table). And the extents of their signal peaks were considered as putative regulatory elements. Third, based on the CHB MAF data in dbSNP database, we only selected the common polymorphisms (MAF > 0.05) that situated within the overlapping parts between the LD block and peaks. Finally, four polymorphisms in high LD with each other (*r*^*2*^ > 0.9) survived after this step-wise analysis. Among them, rs1076394 was chosen as the most potentially functional variant for the genotyping assays, because of its more indicative location in an active enhancer than others’. At the same time, we also tried to replicate the tag SNP rs8180040 in our sample. In Stage 2, the nominal significant SNPs of Stage 1 were further validated. SNPs of both stages were genotyped by a TaqMan real-time polymerase chain reaction (PCR) assay (Applied Biosystems, Foster city, CA). Quality control was preformed by including 5% duplicate samples in blinded fashion, with a concordance rate of 100%.

### Statistical Analysis

The differences in the distributions of gender, age, smoking, drinking status and genotypes between cases and controls were estimated by a χ^2^ test or t-test, where appropriate. The Hardy-Weinberg equilibrium (HWE) in controls was evaluated with a goodness-of-fit χ^2^ test. The odds ratio (ORs) and corresponding 95% confidence intervals (95% CIs) were used to measure the associations between SNPs and CRC susceptibility And they were calculated after adjusting for gender, age, smoking and drinking status under a multivariate logistic regression model. For multiple comparison corrections, a simple procedure (Benjamini and Hochberg) was performed in two stages and combined study to control the false discovery rate^57^. The LD of the candidate SNPs was analyzed using HaploView v4.2^58^. With regard to TCGA and GEO data, the expression differences among three genotypes (TT, TA and AA) of rs8180040 were measured under a linear regression model adjusting the effects of somatic copy number and CpG methylation^43^, and the differences between cancer and normal samples were measured by t-test. The gene-environment interactions were evaluated by a pair-wise analysis under multiplicative^59^ and additive interaction models^60^. All the above statistical analyses were conducted using SPSS Software v20.0 (SPSS, Chicago, Illinois, USA), with the exception that the *P* values of additive interaction were assessed using Stata v11.0 (Stata Corporation, College Station, TX). *P* values in this study were two-sided with a significance criterion of *P* < 0.05.

## Additional Information

**How to cite this article**: Ke, J. *et al*. Identification of a Potential Regulatory Variant for Colorectal Cancer Risk Mapping to 3p21.31 in Chinese Population. *Sci. Rep*. **6**, 25194; doi: 10.1038/srep25194 (2016).

## Supplementary Material

Supplementary Information

## Figures and Tables

**Table 1:** Candidate regulatory SNPs in chromosome locus 3p21.31.

SNP	Position (hg19)	Major/Minor Allele	CHB MAF	Overlapping Peaks	Histone Modifictaions	Cell Line
rs2276854	47276968	T/C	0.49	47276401–47277264	H3K4me1	HCT116
rs807936	47322496	T/C	0.48	47322237–47322873	H3K4me1	HCT116
rs1076394	47322781	G/A	0.48	47322237–47322873	H3K4me1	HCT116
				47322603–47325509	H3K27ac	HCT116
rs807937	47323000	T/G	0.48	47322603–47325509	H3K27ac	HCT116
rs8180040 (tag)	47388947	T/A	0.48	–	–	–

Abbreviations: CHB, Han Chinese in Beijing, China; MAF, minor allele frequency.

**Table 2:** The characteristics of the study population.

	Stage 1				Stage 2				Combine Study			
	Case No. (%)	Control No. (%)	*χ*^*2*^	*P*	Case No. (%)	Control No. (%)	*χ*^*2*^	*P*	Case No. (%)	Control No. (%)	*χ*^*2*^	*P*
Total	767	1397			528	678			1327	2075		
Gender			0.049	0.824			1.625	0.202			0.679	0.410
Male	460 (60.00)	831 (59.48)			316 (59.85)	381 (56.19)			794 (59.83)	1212 (58.41)		
Female	307 (40.00)	566 (40.52)			212 (40.15)	297 (43.81)			533 (40.17)	863 (41.59)		
Age (mean ± SD)	60.42 ± 12.93	61.00 ± 11.99		0.291^a^	59.99 ± 12.34	59.64 ± 13.32		0.635^a^	60.23 ± 12.67	60.56 ± 12.45		0.458^a^
Agegroup			2.540	0.468			3.705	0.295				
≦50	169 (22.06)	333 (23.84)			117 (22.16)	153 (22.57)			294 (22.17)	486 (23.42)	0.737	0.865
51–60	199 (25.98)	388 (27.77)			156 (29.55)	168 (24.78)			364 (27.45)	556 (26.80)		
61–70	211 (27.55)	356 (25.48)			141 (26.70)	201 (29.65)			360 (27.15)	557 (26.84)		
≧71	187 (24.41)	320 (22.91)			114 (21.59)	156 (23.01)			308 (23.23)	476 (22.94)		
Smoking Status			5.955	**0.015**			5.978	**0.014**			10.485	**0.001**
Smoker	307(40.03)	485(34.74)			190(35.98)	199(29.35)			509 (38.42)	684 (32.98)		
Non-Smoker	460(59.97)	911(65.26)			338(64.02)	479(70.65)			816 (61.58)	1390 (67.02)		
Drinking Status			0.850	0.357			0.888	0.346			2.209	0.137
Drinking	224 (29.20)	382 (27.34)			156 (29.60)	184 (27.14)			392 (29.63)	566 (27.28)		
Non-Drinking	543 (70.80)	1015 (72.66)			371 (70.40)	494 (72.86)			931 (70.37)	1509 (72.72)		

Abbreviations: SD, standard deviation.

The nominal significant results were in bold.

^a^*P* value was calculated by the *t* test.

**Table 3:** Association between individual SNP and colorectal cancer risk.

	Stage 1					Stage 2					Combined Study				
	Cases	Controls	OR(95% CI)^a^	*P*^a^	*P-FDR*	Cases	Controls	OR(95% CI)^a^	*P*^a^	*P-FDR*	Cases	Controls	OR(95% CI)^a^	*P*^a^	*P-FDR*
rs8180040															
TT	338	544	1.000			198	208	1.000			536	752	1.000		
TA	324	631	0.827(0.683–1.002)	0.052	0.076	229	322	0.758(0.584–0.982)	**0.036**	0.060	585	953	0.858(0.737–0.999)	**0.048**	0.060
AA	103	218	0.764(0.582–1.003)	0.052	0.076	95	143	0.723(0.521–1.002)	0.051	0.064	198	361	0.773(0.629–0.950)	**0.015**	**0.025**
Dominant			0.811(0.677–0.970)	**0.022**	0.825			0.747(0.586–0.952)	**0.018**	0.090			0.835(0.724–0.963)	**0.013**	**0.033**
Recessive			0.839(0.651–1.082)	0.176	0.176			0.847(0.633–1.133)	0.264	0.264			0.840(0.695–1.015)	0.071	0.071
Additive			0.861(0.758–0.979)	**0.022**	0.825			0.837(0.713–0.983)	**0.030**	0.075			0.875(0.793–0.966)	**0.008**	**0.040**
rs1076394															
GG	309	490	1.000			197	205	1.000			507	695	1.000		
GA	336	639	0.835(0.687–1.015)	0.070	0.070	230	322	0.758(0.584–0.984)	**0.037**	0.062	597	961	0.850(0.728–0.991)	**0.038**	**0.038**
AA	122	267	0.723(0.559–0.936)	**0.014**	**0.035**	92	139	0.716(0.514–0.996)	**0.047**	0.059	214	406	0.723(0.591–0.885)	**0.002**	**0.005**
Dominant			0.802(0.669–0.963)	**0.018**	**0.030**			0.745(0.584–0.951)	**0.018**	0.090			0.812(0.703–0.938)	**0.005**	**0.008**
Recessive			0.797(0.629–1.008)	0.059	0.074			0.840(0.625–1.127)	0.245	0.245			0.793(0.660–0.951)	**0.013**	**0.016**
Additive			0.847(0.748–0.960)	**0.009**	**0.045**			0.833(0.708–0.980)	**0.027**	0.068			0.850(0.771–0.938)	**0.001**	**0.005**

Abbreviations: OR, Odds ratio; 95% CI, 95% confidence interval; FDR, false discovery rate.

Bold values indicated *P* < 0.05.

^a^Data were calculated by logistic regression model after adjusting for sex, age group, smoking and drinking status.

**Table 4:** Expression correlation between rs8180040 and flanking 1 Mb genes.

Gene	Correlation R2	Correlation P	Correlation P-FDR
CCDC12	3.091E-02	4.952E-03	1.387E-01
NME6	1.882E-02	2.884E-02	4.038E-01
PTH1R	8.257E-03	1.487E-01	1.000
CDC25A	8.041E-03	1.542E-01	1.000
LTF	7.674E-03	1.639E-01	9.180E-01
MYL3	7.498E-03	1.689E-01	7.881E-01
LRRC2	6.062E-03	2.162E-01	8.649E-01
DHX30	5.517E-03	2.382E-01	8.336E-01
CAMP	5.229E-03	2.509E-01	7.805E-01
CCR2	4.858E-03	2.684E-01	7.517E-01
SMARCC1	3.693E-03	3.348E-01	8.521E-01
RTP3	2.954E-03	3.883E-01	9.061E-01
SETD2	2.748E-03	4.055E-01	8.734E-01
CCRL2	2.407E-03	4.362E-01	8.725E-01
KIF9	2.382E-03	4.387E-01	8.189E-01
NBEAL2	1.133E-03	5.933E-01	1.000
TDGF1	1.117E-03	5.959E-01	9.815E-01
PRSS50	1.071E-03	6.037E-01	9.390E-01
NRADDP	6.032E-04	6.969E-01	1.000
MAP4	5.606E-04	7.073E-01	9.902E-01
PTPN23	4.698E-04	7.310E-01	9.747E-01
KLHL18	3.567E-04	7.645E-01	9.730E-01
ALS2CL	3.426E-04	7.691E-01	9.363E-01
ZNF589	3.120E-04	7.794E-01	9.093E-01
SCAP	3.056E-04	7.816E-01	8.754E-01
TMIE	2.218E-04	8.133E-01	8.758E-01
CSPG5	5.248E-05	9.085E-01	9.422E-01
CCR5	4.253E-05	9.176E-01	9.176E-01

Abbreviations: FDR, false discovery rate.

The nominal significant results were in bold.
