# Supplementary material for: Identification of a Potential Regulatory Variant for Colorectal Cancer Risk Mapping to 3p21.31 in Chinese Population
Source: Sci Rep. 2016 Apr 28;6:25194. doi: 10.1038/srep25194 (PMC4848543; doi:10.1038/srep25194)
Supplement: Supplementary Information [file srep25194-s1.pdf]

Identification of a Potential Regulatory Variant for Colorectal Cancer Risk Mapping to 3p21.31 in Chinese Population

Juntao Ke[1], Jiao Lou[1], Rong Zhong[1], Xueqin Chen[1], Jiaoyuan Li[1], Cheng Liu[1], Yajie Gong[1], Yang Yang[1], Ying Zhu[1], Yi Zhang[1], Jiang Chang[1], Jing Gong[1*]

[1] State Key Laboratory of Environment Health (Incubation), MOE (Ministry of Education) Key Laboratory of Environment & Health, Ministry of Environmental Protection Key Laboratory of Environment and Health (Wuhan), School of Public Health, Tongji Medical College, Huazhong University of Science and Technology, Wuhan, China

[*]**Correspondences:**

E-mail: gongj@hust.edu.cn (JG)

Table S1. ChIP-seq datasets downloaded from UCSC integrating Encode data

Cell line	Mark	Dataset
Hct116	H3k4me1	wgEncodeSydhHistoneHct116H3k04me1UcdPk.narrowPeak
Hct116	H3k27ac	wgEncodeSydhHistoneHct116H3k27acUcdPk.narrowPeak
Hct116	H3k4me3	wgEncodeUwHistoneHct116H3k4me3StdPkRep1.narrowPeak
		wgEncodeUwHistoneHct116H3k4me3StdPkRep2.narrowPeak
Caco2	H3k27me3	wgEncodeUwHistoneCaco2H3k27me3StdPkRep1.narrowPeak
		wgEncodeUwHistoneCaco2H3k27me3StdPkRep2.narrowPeak
Caco2	H3k36me3	wgEncodeUwHistoneCaco2H3k36me3StdPkRep1.narrowPeak
		wgEncodeUwHistoneCaco2H3k36me3StdPkRep2.narrowPeak
Caco2	H3k4me3	wgEncodeUwHistoneCaco2H3k4me3StdPkRep1.narrowPeak
		wgEncodeUwHistoneCaco2H3k4me3StdPkRep2.narrowPeak

Table S2. Interaction analysis between smoking and rs1076394 associated with CRC risk

		Stage 1		Stage 2		Combined study	
Smoking stusus	Genotype	Case/Control	OR (95%CI)[a]	Case/Control	OR (95%CI)[a]	Case/Control	OR (95%CI)[a]
Non-smoker	GG	183/325	1.000	120/144	1.000	303/469	1.000
	GA+AA	277/586	0.838 (0.665-1.056)	230/330	0.840 (0.625-1.129)	507/916	0.856 (0.714-1.025)
Smoker	GG	126/164	1.441 (1.048-1.983)	78/61	1.566 (0.986-2.488)	204/225	1.457 (1.123-1.890)
	GA+AA	181/320	1.075 (0.809-1.428)	131/121	1.155 (0.769-1.736)	302/451	1.079 (0.857-1.359)
P_{mult}[a]			0.822		0.724		0.853
P_{add}			0.826		0.791		0.891

P_{mult} was calculated using the multiplicative interaction term.

P_{add} was calculated using the additive interaction model.

[a] Data were calculated by logistic regression model after adjusting for gender, age group and drinking status.
